# Implementing MyChoice^®^ CDx HRD testing for the Nordics: lessons from 2021 to 2023

**DOI:** 10.2340/1651-226X.2024.34139

**Published:** 2024-03-14

**Authors:** Lea Milling Korsholm, Verena Broecker, Mansoor Raza Mirza, Maria Rossing

**Affiliations:** a Center for Genomic Medicine, Copenhagen University Hospital – Rigshospitalet, 2100 Copenhagen, Denmark; b Department of Clinical Pathology, Sahlgrenska University Hospital, 413 45 Gothenburg, Sweden; c Department of Oncology, Rigshospitalet, Copenhagen University Hospital – Rigshospitalet, 2100 Copenhagen, Denmark; d Department of Clinical Medicine, University of Copenhagen, Copenhagen, Denmark

**Keywords:** Ovarian, cancer, HRD, biomarker, Myriad, PARPi, BRCA

## Abstract

**Background:**

Assessment of homologous recombinant deficient (HRD) phenotypes is key for managing Poly (ADP-ribose) polymerase inhibitor (PARPi) treatment. To accommodate the need for a validated HRD platform and enhance targeted treatment of ovarian cancer patients, a Nordic core facility for the myChoice^®^ CDx platform was established in Denmark.

**Materials and methods:**

Comparative calculations and statistics are based on information from test requisitions and results (Genome Instability Score [GIS], *BRCA* status and combined HRD status) obtained from ovarian and breast cancer samples submitted for HRD-testing by myChoice^®^ CDx through the Nordic core facility in the 2-year period.

**Results:**

Copenhagen University Hospital received 1,948 requisitions during the 2-year period. Conclusive results were obtained in 89% of the tests, while 7% were inconclusive due to the lack of GIS and 4% were not able to be analysed. Comparing the conclusive HRD status results across countries, Sweden had the highest percentage of HRD positives (38%) compared to Denmark, Norway, and Finland (28–32%).

**Interpretation:**

The myChoice^®^ CDx Nordic core facility has been well received among the Nordic countries and provides new insights on the influence of national guidelines on HRD testing. Overall, we experienced an efficient turnaround time and a high fraction of conclusive results. Interestingly, prior somatic *BRCA* testing is redundant when assessing HRD status through myChoice^®^ CDx test since somatic *BRCA* screening is already a significant component of the myChoice^®^ CDx test. Thus, it should be considered to omit prior somatic *BRCA* testing to ensure a rationalised HRD diagnostic flow optimised for clinical use.

## Introduction

The clinical development of Poly (ADP-ribose) polymerase (PARP) – inhibitor (PARPi) [1, 2] has revolutionised the management of ovarian cancer (including ovarian, tubal- and peritoneal cancer) patients who suffer from a homologous recombinant deficient (HRD) tumour [3]. HR-deficiency occurs due to the loss of function of homologous recombination (HR) repair pathways, often caused by mutations in essential HR repair genes, such as the *Breast Cancer* (*BRCA*) *1/2* genes [4]. The synthetic lethal relationship between inhibition of PARP enzymes and *BRCA1/2* loss of function is rooted in DNA damage accumulation [5]. PARP is essential for repair of single strand DNA breaks, and inhibition of PARP therefore leads to break accumulation, resulting in the formation of double strand DNA breaks. Thus, the combination of HR repair pathway deficiencies and PARPi treatment will result in targeted cell death due to the persistence of double strand DNA breaks in the genome [6, 7].

It is by now well-established that PARPi could also be efficacious in non-*BRCA* mutated, HRD tumours [8], and several clinical trials have proved significantly increased progression-free survival upon PARPi treatment among non-*BRCA*-mutated HRD patients [9, 10]. HRD tumours, devoid of the HR repair pathway, characteristically display heavily scarred genomes. This hallmark, and its detection, is currently used to identify which ovarian cancer patients will likely benefit from PARPi treatment.

Currently, the two FDA-approved diagnostic tests widely in use for HRD-detection are myChoice® CDx from Myriad Genetics (Salt Lake City, Utah, USA) and FoundationOne® CDx from Foundation Medicine (Cambridge, Massachusetts, USA). Both tests use DNA isolated from formalin-fixed paraffin embedded (FFPE) tumour tissue specimens and next generation sequencing. They detect single nucleotide variants, insertions and deletions, and large rearrangements in protein coding regions and intron/exon boundaries of HR-associated genes to determine HRD status in patients with ovarian cancer. In addition to the HR-associated gene panels, the FoundationOne CDx test assesses detection of genomic loss of heterozygosity (LOH) [11], whereas the myChoice® CDx platform estimates a genomic instability score (GIS) which is an algorithmic measurement of LOH, telomeric allelic imbalance (TAI), and large-scale state transitions (LST) [12].

GIS status is positive when GIS ≥ 42, negative when GIS < 42 or inconclusive if the algorithm did not return with a score. *BRCA* result is either positive, upon detection of a pathogenic or likely pathogenic *BRCA* mutation, or negative, either due to no variant detection or if the detected variant is classified as variant of uncertain significance (VUS). The combined HRD status is positive when GIS ≥ 42 or a likely pathogen *BRCA* mutation is identified, and the combined HRD status is negative when GIS < 42 and no pathogen *BRCA* mutation is detected, or inconclusive, if the GIS was inconclusive and no pathogen *BRCA* mutation is detected.

Today, PARPi is used for the management of patients with ovarian, breast, pancreatic or prostate cancers, depending on the displayed HRD phenotypes [13,14]. However, only ovarian cancer can be classified as HRD-positive based on genomic LOH and GIS score alone [14].

To accommodate the need for a validated HRD platform and to enhance targeted treatment of ovarian cancer patients in the Nordic countries, a collaboration between the Center for Genomic Medicine (GM), Rigshospitalet, Copenhagen University Hospital, Denmark and Myriad Genetics, Salt Lake City, Utah, USA, was initiated, with the purpose of establishing a partner-lab and Nordic core facility for the myChoice® CDx platform. In addition, the partner-lab would handle all legal and practical concerns, such as intercontinental data and transfer agreements. In the implementation phase, GM coordinated myChoice® CDx requisitions from all Nordic countries, forwarded them to the Myriad site laboratory in Salt Lake City and distributed the subsequent HRD test results. Here, we reveal the insights and statistics from the first 2 years of implementing Myriad HRD-testing in the Nordic countries and discuss the common lessons we gained.

## Patients and methods

### Study cohort

Included in this study are FFPE samples, obtained from ovarian and breast cancer tumours, submitted for HRD-testing by myChoice® CDx (Myriad Genetics) in the period July 21st, 2021 to July 20th, 2023, through the Nordic core facility. The samples were obtained from requisition sites in Denmark, Sweden, Norway, Finland, Iceland, Lithuania, Estonia, and Germany. When requesting a MyChoice® CDx test, the local pathologist was required to report the estimated tumour percentage in the FFPE samples, as the MyChoice® CDx test is validated to tumour level above 30%. The testing procedure and data processing agreement (DPA) was approved by The Capital Region of Denmark (dated July 5th, 2021), Copenhagen University Hospital and Myriad Genetic, Inc.

### Data collection

All data points from the requisition sites and myChoice® CDx analysis results (GIS score, *BRCA* status and combined HRD status) were assembled anonymously.

### Statistical analyses

Figures and statistical analyses were performed with Excel (Microsoft Excel 2016), SPSS software (IBM SPSS Statistics 28.0.0.0) and GraphPad Prism software (GraphPad Prism version 9.4.1). A *p*-value of <0.0001 was considered significant. The chosen statistical test for each analysis is indicated in figure legends. Figures were combined and visually adjusted using Illustrator (Adobe Illustrator version 27.11).

## Results

**Figure 1:**
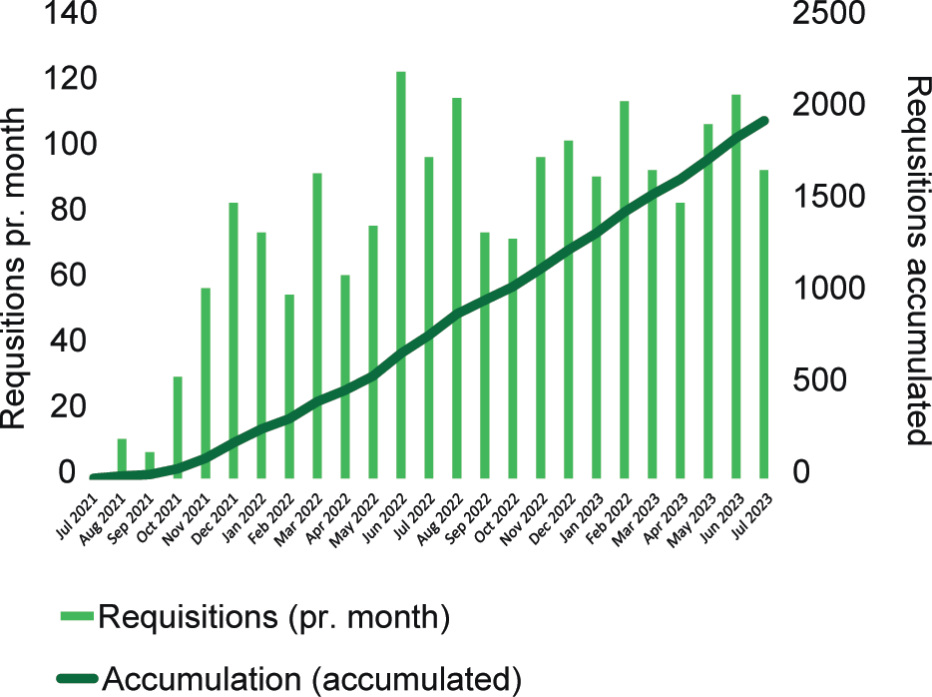
Monthly requisitions (bar chart) and running accumulation (graph) of myChoice® CDx requisitions received from all participating countries from July 21st, 2021 to July 20th, 2023, at the Nordic core facility. Final accumulated test requisitions pr. July 20th, 2023, is *n* = 1,948.

In the implementation phase of the partner-lab and Nordic core facility for the myChoice® CDx platform, GM coordinated test requisitions from all Nordic countries, forwarding them to the Myriad site laboratory in Salt Lake City. The first requisition was obtained on July 21st, 2021, and to evaluate exactly 2 years of progress, the last test included in the analysis was obtained on July 20th, 2023. In total, GM received 1,948 requisitions during the 2-year period. Monthly requisitions and the cumulative number of requisitions are depicted in Figure 1. The average time from receiving the sample at the GM lab to reporting of the test results was 22 days.

**Figure 2:**
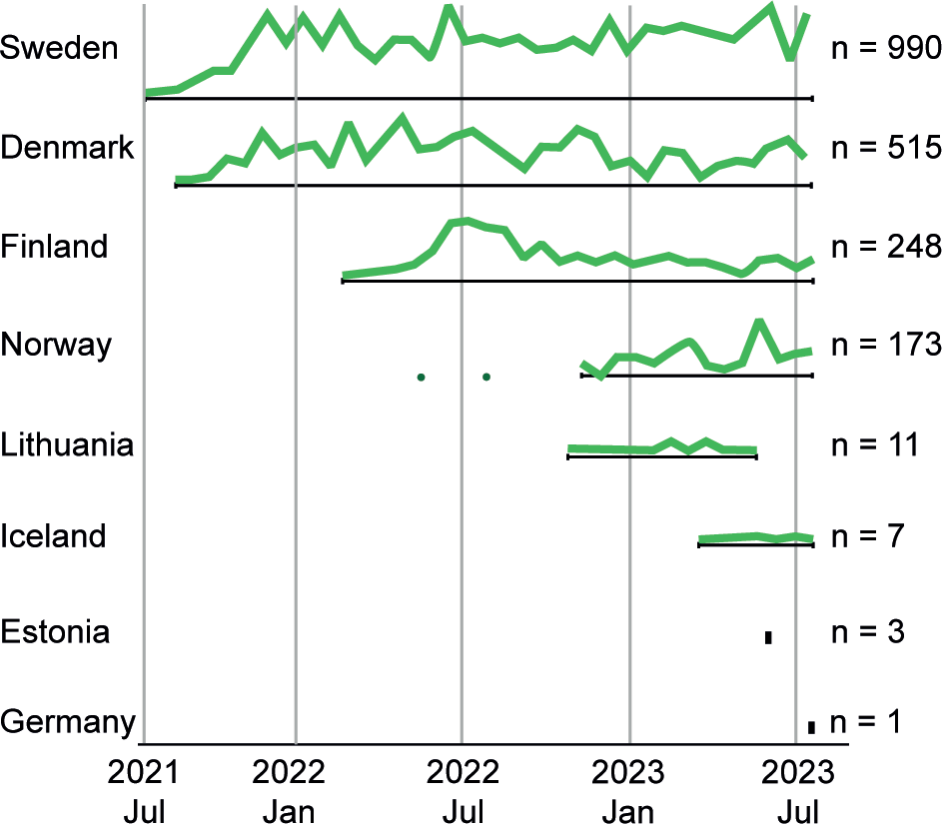
Illustrative representation of the relative distribution of myChoice® CDx requisitions from the countries participating in the implementation of the partner-lab and Nordic core facility from July 21st, 2021 to July 20th, 2023. Total number of requisitions are annotated for each country (*n*).

The five Nordic countries, Denmark, Sweden, Norway, Finland, and Iceland, along with Estonia, Lithuania and Germany requested testing through the Nordic core facility, via sending samples for myChoice® CDx analysis to GM at Copenhagen University Hospital. The distribution of test requisitions over time is depicted in Figure 2 and the accumulated number of test requisitions is listed for each country: Sweden = 990, Norway = 173, Estonia = 3, Lithuania = 11, Germany = 1, Iceland = 7, Finland = 248 and Denmark = 515.

**Figure 3:**
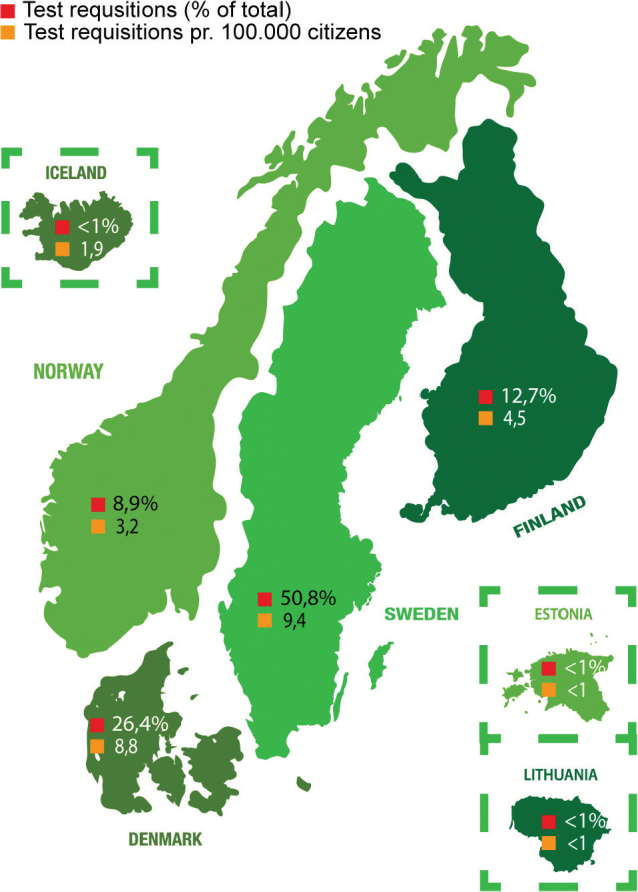
Schematic overview of the countries taking advantage of the Nordic core facility and forwarding myChoice® CDx requisitions to GM. Symbolised with red square is the percentage of test requisitions from each individual country out of the total number of requisitions (*n* = 1,948). Symbolised with yellow square is the number of test requisitions pr. 100,000 citizens in the country.

To further examine the distribution of test requisitions among the participating countries, the percentage of test requested from each individual country out of the total number of test requisitions received at GM (*n* = 1,948) is shown in Figure 3. Likewise, the number of test requisitions pr. 100,000 citizens, is calculated for a more comprehensive overview. Germany is not included in the schematic overview (Figure 3), as the German site only forwarded a single sample in the 2-year period. Schematic overviews depicting test requisitions from the individual regions of Denmark, Norway, Sweden, and Finland are shown in Supplementary Figure 3A–D.

**Figure 4:**
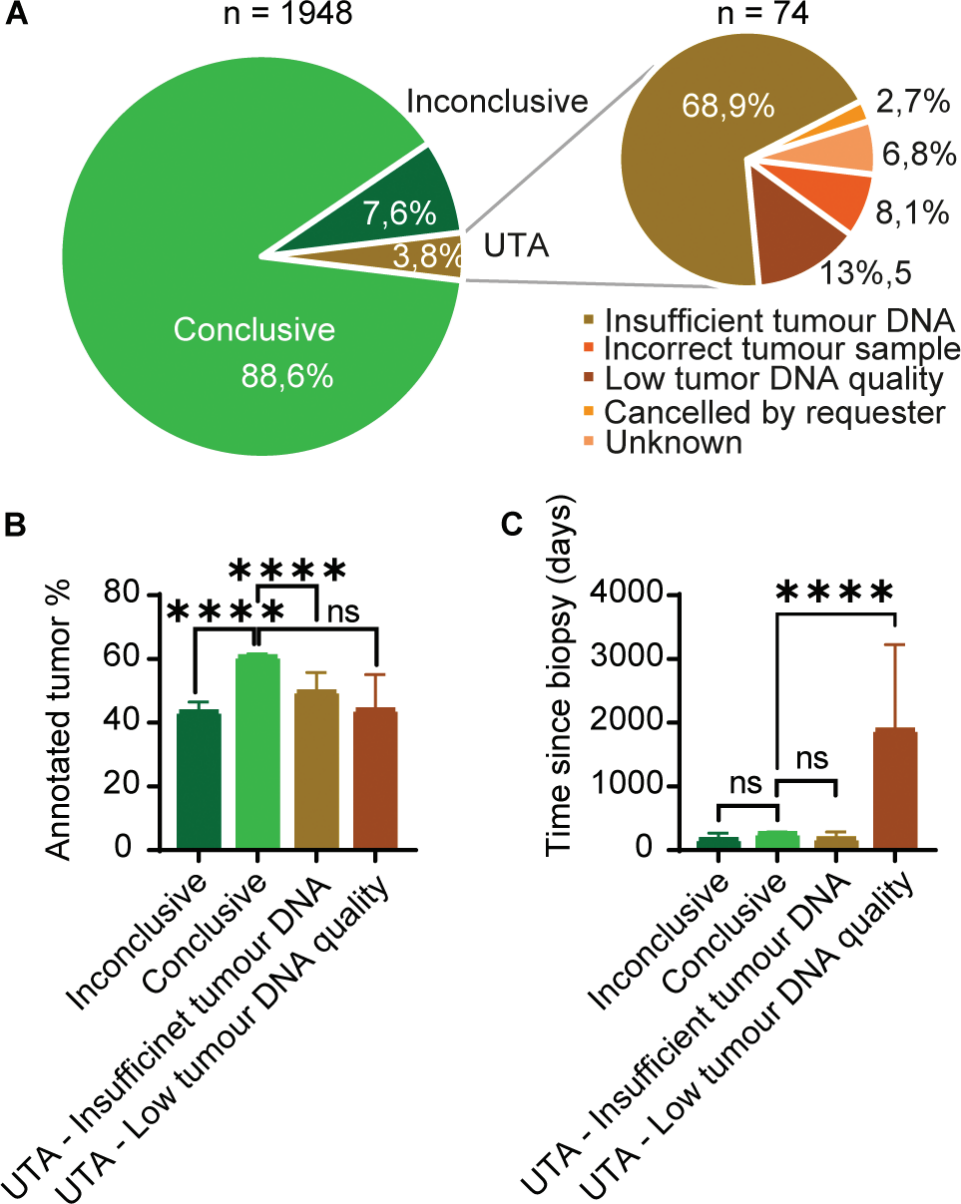
The success rate of myChoice® CDx tests. (A) Circle diagram depicting the success rate of test analysis. Of the total test requisitions (*n* = 1,948) 1,726 yielded a conclusive HRD status result, 148 were inconclusive and 74 were unable to be analysed (UTA). The cause for UTA: incorrect tumour sample (*n* = 6), low tumour DNA quality (*n* = 10), insufficient tumour DNA (*n* = 51), the requisition was cancelled (*n* = 2), and unknown (*n* = 5). (B): Graph depicting the distribution of annotated tumour percentage (mean with 95% confidence interval) between conclusive (*n* = 1,726), inconclusive (*n* = 148) test results and UTA due to insufficient tumour DNA (*n* = 51) and UTA due to low tumour DNA quality (*n* = 10). Unpaired 2-tailed t-test, **** = *p* < 0.0001, not significant (ns) = *p* > 0.05. (C) Graph depicting the distribution of time since biopsy (mean with 95% confidence interval) between conclusive, inconclusive and UTA test results. Unpaired 2-tailed *t*-test; **** = *p* < 0.0001, not significant (ns) = *p* > 0.05.

The success rates of myChoice® CDx tests are summarised in Figure 4. Of the total tests (*n* = 1,948), 88.6% yielded a conclusive result (*n* = 1,726), classifying the tumour as either HRD status positive or negative. 7.6% of myChoice® CDx tests (*n* = 148) generated an inconclusive result, meaning that the genomic instability algorithm did not yield a score and no pathogenic *BRCA* mutations were detected. The remaining 3.8% of the myChoice® CDx tests requested (*n* = 74) did not yield results because they could not be analysed (unable to analyse [UTA]). A variety of reasons lie behind the UTA samples, the main reasons being an insufficient amount of tumour DNA (*n* = 51), too low tumour DNA quality (*n* = 10) and requests made on an incorrect tumour sample (*n* = 6). Lastly, two tests were cancelled during analysis and five were UTA due to unknown reasons (Figure 4A). As previously noted, the myChoice® CDx test is validated to tumour level above 30%. When comparing the annotated tumour percentage (Figure 4B), inconclusive samples had a mean of 43% tumour cells, which was significantly lower (*p* < 0.0001) compared to the conclusive samples with a mean of 61%. Likewise, there was a significant difference between samples with conclusive results and UTA due to insufficient tumour DNA with a mean tumour percentage of 49% (*p* < 0.0001). Samples with UTA results due to low tumour DNA quality also had a mean tumour percentage of 49%; however, the difference was not statistically significant, likely due to the low number of samples.

Upon requisition of a myChoice® CDx test, the local pathologist also reports the date when the tumour sample was obtained. When comparing the sample age (Figure 4C), no significant difference was observed between samples yielding a conclusive and inconclusive result (*p* = 0.2294). The mean age of biopsies with an inconclusive result was 202 days and for conclusive results 261 days. Samples that were UTA due to insufficient tumour DNA had a mean biopsy age of 181 days and were not significantly different from the samples with conclusive results (*p* = 0.3053). On the contrary, samples that were UTA due to low tumour DNA quality had a significantly higher mean biopsy age of 1,885 days, compared to conclusive results (*p* < 0.0001). Out of the total numbers of HRD test requisitions (*n* = 1,948), 26 sample analysis were repeated one time and one sample analysis was repeated two times due to UTA or inconclusive results. After repeat analysis, 13/26 samples obtained a conclusive result after the first repeat, and one sample after the second repeat. The remaining 13 samples did not obtain conclusive results and their analysis remained inconclusive (*n* = 10) or UTA (*n* = 3). The three samples that remained UTA were all due to insufficient tumour DNA. Thus, the 74 samples that resulted in a final status of UTA were excluded from the downstream analysis of myChoice® CDx test results.

**Figure 5:**
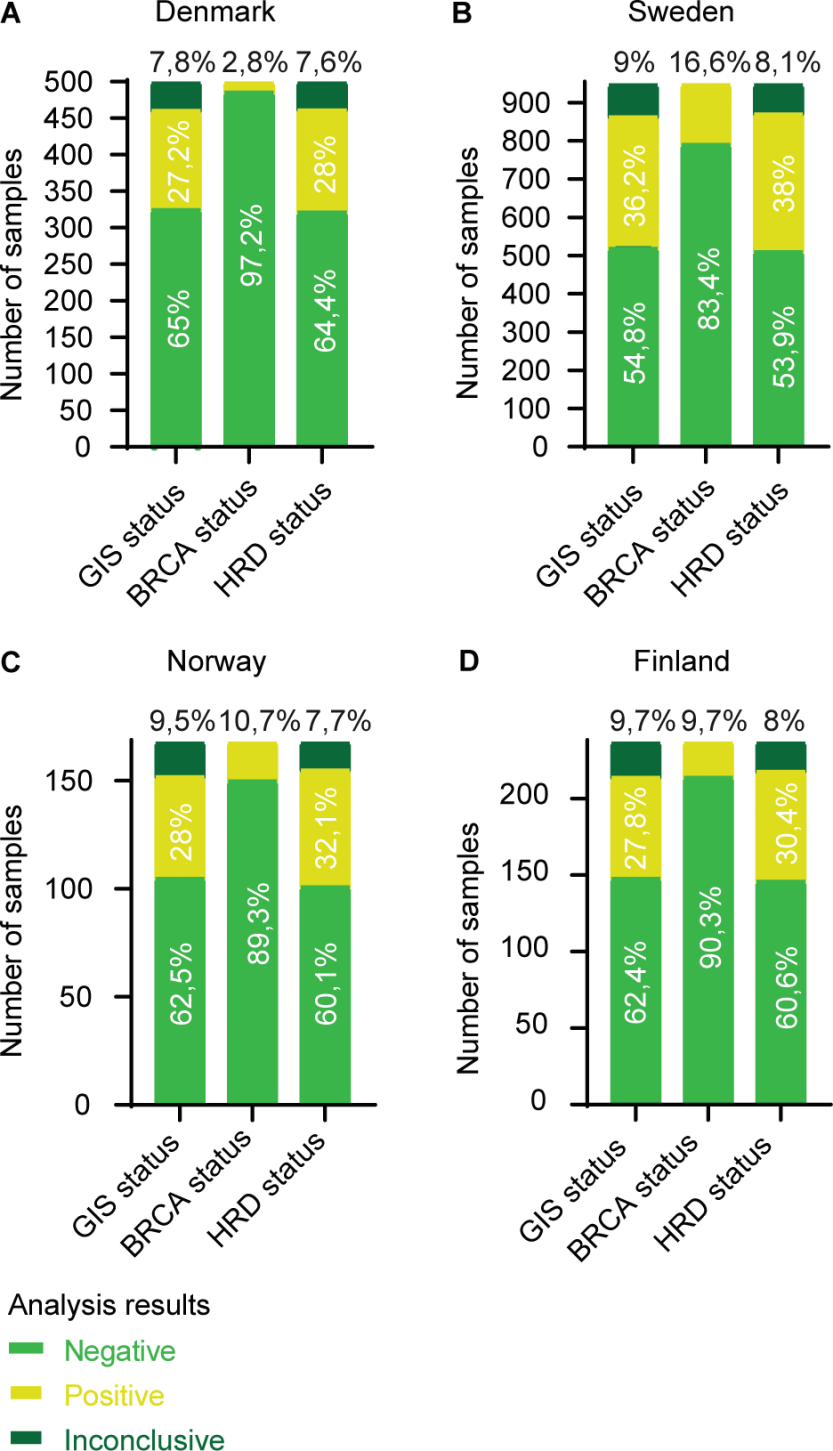
Number of myChoice® CDx analysis results from (A) Denmark (*n* = 500), (B) Sweden (*n* = 948), (C) Norway (*n* = 168) and (D) Finland (*n* = 237). The number of test results are separated in the status categories; GIS status: GIS ≥ 42 = positive and GIS < 42 = negative. *BRCA* mutation status: pathogenic *BRCA* mutation = positive and no pathogenic *BRCA* mutation = negative. Thus, HRD positive status is a result of either a GIS ≥ 42 and/or a pathogen *BRCA* mutation. Result distribution within each category is annotated in percentage.

To get a more comprehensive view of the distribution of myChoice® CDx test results, we compared the results from GIS score, *BRCA* results and the combined HRD status among the four main contributing countries: Denmark (Figure 5A), Sweden (Figure 5B), Norway (Figure 5C) and Finland (Figure 5D). The remaining countries were excluded from the analysis due to the low number of requisitions (Lithuania = 11, Estonia = 3, Iceland = 6, and Germany = 1).

The percentage of samples with an inconclusive GIS are comparable between Norway, Sweden, and Finland, with approximately 9–10%, and 7.8% for Denmark. The percentage of samples with a positive GIS is comparable between Denmark, Norway, and Finland, with approximately 27–28%, whereas Sweden was substantially higher with 36.2%. A similar pattern is repeated when assessing the *BRCA* status. Sweden had the highest *BRCA* positive percentage with 16.6%. Norway and Finland both had approximately 10%, and Denmark had the lowest percentage of *BRCA* positive samples with only 2.8%. Considering the HRD status overall, the inconclusive samples accounted for approximately 8% across all four countries. Sweden had the highest percentage of HRD positive results (38%), whereas Denmark, Norway and Finland were in the range of 28–32%. Sweden was the only country requesting HRD-testing on breast cancer samples (*n* = 13), of which eight were HRD positive (four of them *BRCA* positive) and five HRD negative.

## Discussion and perspectives

To accommodate the need for a validated HRD platform for targeted treatment of ovarian cancer patients in the Nordic countries, we initiated a collaboration between Copenhagen University Hospital and Myriad Genetics. The collaboration has been well received, and with the culmination of the first 2 years of broadly implementing the Myriad HRD-test, we see an increased employment from the Nordic and neighbouring countries. Furthermore, we have experienced a relatively high efficiency of the GM based HRD-testing initiative, with a mean turnaround time of approximately 3 weeks and a rate of inconclusive results below 10%.

The four main Nordic participators are responsible for 98.8% of the total myChoice® CDx test requisitions (99.2% including Iceland). Considering the different sizes of populations, we calculated the number of test requisitions pr. 100,000 citizens and found Denmark and Sweden to range at the top of the list, with approximately 9 HRD – test requisitions pr. 100,000 citizens, while Iceland, Norway and Finland had approximately two, three and five, respectively. The number of requisitions is also reflected in the participation timeline from the contributing countries; Sweden and Denmark submitted the initial samples in 2021, with Finland and Norway following in 2022, and Iceland in 2023. Distribution of requisitions further differs substantially within each country, depending on the specific region. In Denmark, ‘Region Midtjylland’ and ‘Region Hovedstaden’ had approximately 12 test requisitions pr. 100,000 citizens, while ‘Region Nordjylland’ and ‘Region Sjælland’ notably had only 3.3 and <1. This distribution indicates that the hospitals in the largest cities are taking advantage of the validated HRD test opportunity as recommended in the national guidelines. The same pattern is observed in Sweden; however, here it should also be noted that the less populated ‘Övre Norrland’, with 9.3 requisitions pr. 100,000 citizens, is highly compatible with the Stockholm area having 9.8 requisitions pr. 100,000 citizens. Interestingly, a different pattern is observed in Norway, where the less populated ‘Helse-Nord’, with 4.6 test requisitions, is having the highest numbers of requisitions pr. 100,000 citizens, as opposed to the larger hospitals in Stavanger and Bergen located in ‘Helse-Vest’, which only have 1.5 requisitions pr. 100,000 citizens. In Finland, one of the regions with lowest number of requisitions pr. 100,000 citizens is HYKS region (including Helsinki), with 2.9 requisitions, while the area with the highest number is TAYS (including the third biggest city Tampere), with 9.1 requisitions pr. 100,000 citizens. Taken together, we recognised an unequal distribution of test requisitions across regions and countries.

The success rate of the myChoice® CDx analysis is overall high, with only 7.6% of the requisitions yielding an inconclusive result. The main reason for inconclusive results could be linked to a low tumour percentage. Even though the mean tumour percentage for inconclusive samples was above the required 30%, the analysis was not optimal. Likewise, samples with UTA results, due to insufficient tumour DNA, also have a significantly lower tumour percentage compared to samples with conclusive test results, although they had a mean percentage above 30%. These observations emphasise the importance of a high tumour percentage for the analysis outcome and that pathologists potentially overestimate the tumour percentage in samples before forwarding them for analysis. To assess the influence of biopsy age on the HRD test results, we compared time measured in days since biopsy across the result categories; conclusive, inconclusive and UTA due to insufficient tumour DNA and UTA due to low tumour quality. Biopsy age did not significantly influence the inconclusive test results. Only samples with UTA results due to low tumour DNA quality had a significantly higher biopsy age, compared to the three other result categories. When assessing the subgroup of repeated tests due to the first results being inconclusive or UTA, only about half of the repeated tests resulted in a final conclusive result, while the other half remained inconclusive or UTA. Overall, these observations highlight the importance of using good quality samples with a high tumour percentage for the test requisitions to ensure an efficient sample-to-result flow.

We observed a substantial difference in the distribution of HRD positive test results across the four main Nordic participators. Denmark, Norway, and Finland had an approximately 28–32% positive HRD status rate, compared to Sweden having 38% positive HRD results. Looking further into the two components reflected in the combined HRD status, GIS and *BRCA* mutation status, we observed similar patterns of differences in the distribution of myChoice® CDx test results. Assessing the *BRCA* test results, Sweden had the highest portion of positive *BRCA* samples (16.6%) as opposed to Denmark who only had 2.8% positive *BRCA* test results. Norway and Finland both had approximately 10% of samples with a positive *BRCA* test result. The differences are likely caused by the national and local *BRCA* testing strategies and recommendations within the Nordic countries. The European Society of Medical Oncology (ESMO) guidelines recommend *BRCA* mutational screening of all ovarian cancers, besides mucinous ovarian cancer [15, 16], as well as recognising that HRD tests are beneficial for predicting response to PARPi [17]. In Denmark, the guidelines regarding HRD testing for ovarian cancer patients were updated in 2022 (http://www.dgcg.dk/images/retningslinier/Ovariecancer/DGCG_BRCA12_HRD_testning_ovariecancer_V.1.0_AdmGodk_0201235719.pdf) and state that all tissue samples should be screened for somatic *BRCA* mutations and if wildtype (wt), the sample should undergo HRD testing using the myChoice® CDx platform. Although all the Danish myChoice® CDx test requisitions are performed on samples which have been *BRCA* screened earlier and found negative, 27.2% are still found to have a positive GIS. In Sweden, a substantially higher GIS positive fraction is present (36.2%), reflecting that the Swedish guidelines do not require pre-screening for somatic *BRCA* mutations, before HRD testing (https://kunskapsbanken.cancercentrum.se/globalassets/cancerdiagnoser/gynekologi/aggstockscancer/nationellt-vardprogram-aggstockscancer.pdf). Interestingly, the fraction of GIS positive results from Finland (27.8%) and Norway (28%) were very similar to the Danish results, although their fraction of *BRCA* positive samples was a lot higher than Denmark. Overall, prior somatic *BRCA* testing does not diminish the utility of the myChoice® CDx test when assessing HRD status. On the contrary, HRD testing fills a gap, as a substantial proportion of patients were detected to be eligible for PARPi-therapy on the grounds of having high GIS despite being negative for somatic BRCA-mutation. Furthermore, considering that somatic *BRCA* testing is already a significant component of the myChoice® CDx test, prior *BRCA* screening seems excessive. To optimise the resources allocated to HRD testing, it should therefore be considered if prior somatic *BRCA* testing should be omitted, without compromising the final HRD status results.

In line with ESMO acknowledging the clinical benefits of HRD tests, while also requesting further validation, additional validation of the myChoice® CDx test is currently ongoing. Hence, two test requisitions, both from Norway, are a part of the newly started NSGO-CTU/HERO trial, a prospective observational study characterising epithelial ovarian cancer patients in terms of HRD status. Additionally, 21 test requisitions were a part of the DOVACC trial (NCT04742075) evaluating the efficacy of UV1-olaparib-durvalumab combination as maintenance therapy after platinum combination therapy for *BRCA* wt patients with relapsed ovarian cancer. Of these, one was from Germany, two from Finland, seven from Norway and 11 from Lithuania.

Overall, we have provided a comprehensive study on the first 2 years of implementing HRD testing through the Nordic core MyChoice^®^ CDx facility. One obvious drawback of the study has been the lack of access to raw data generated by Myriad, e.g., from samples where the GIS was UTA and thus, we could not investigate the specific reasons for failed testing. However, the centralisation of both requisitions and the myChoice^®^ CDx results across, and beyond, the Nordic countries enabled a large-scale study and provided new insights both regarding efficiency and pitfalls of the myChoice^®^ CDx test, as well as the influence of national guidelines on HRD testing.

The Myriad Genetics partner-lab at the Center for Genomic Medicine at Copenhagen University Hospital is now licensed with sample flow and patient data handled according to an extended DPA. Altogether, the Nordic core facility is reducing both cost as well as legal and practical concerns and thus enabling myChoice® CDx testing in the Nordics a feasible option for HRD-testing in the routine diagnostic setting.

## Disclosure statement

MR reports personal fees for advisory board for MSD and talks for MSD and GSK.

MRM reports the receipt of personal fees from AstraZeneca, Pfizer and Clovis Oncology, personal fees from Biocad, Geneos, Genmab, Oncology Venture, and Merck, personal fees and other from Karyopharm Therapeutics, Sera Prognostics, Seattle Genetics, Sotio, and ZaiLab outside the submitted work.

LMK and VB report no competing interests.

## Data availability statement

The data that support the findings of this study are available from the corresponding author, [MR], upon reasonable request.

## Ethical statement

Ethical approval was waived by the local ethics committee since all data points were anonymised.

## Supplementary Material

Implementing MyChoice^®^ CDx HRD testing for the Nordics: lessons from 2021 to 2023
