# Supplementary material for: Implementing MyChoice® CDx HRD testing for the Nordics: lessons from 2021 to 2023
Source: Acta Oncol. 2024 Mar 14;63:34139. doi: 10.2340/1651-226X.2024.34139 (PMC11332506; doi:10.2340/1651-226X.2024.34139)
Supplement: Implementing MyChoice® CDx HRD testing for the Nordics: lessons from 2021 to 2023 [file AO-63-34139-s1.pdf]

Supplementary material has been published as submitted. It has not been copyedited or typeset by Acta Oncologica.

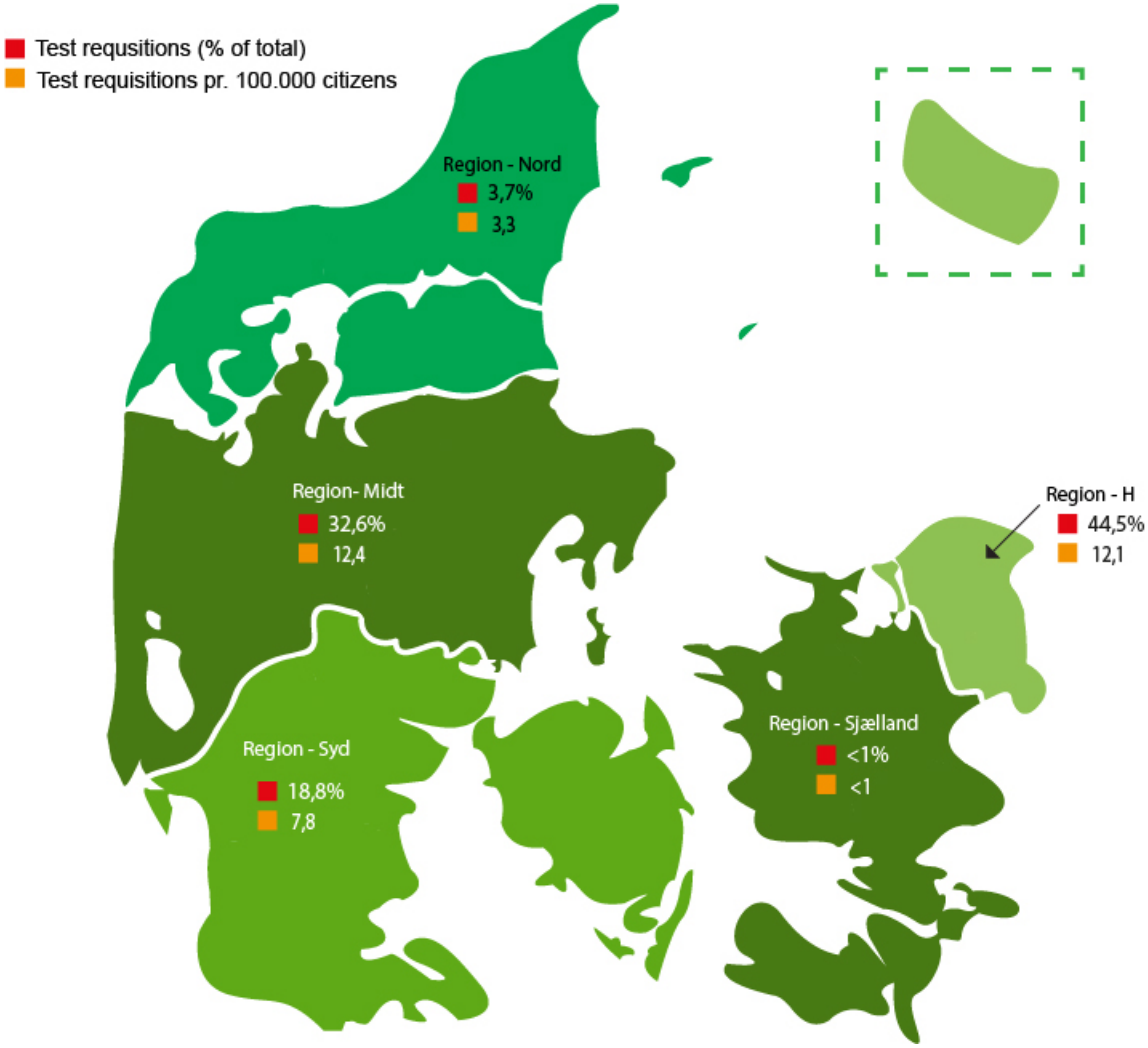

Supplementary figure 3A Schematic overview of the myChoice® CDx requisitions send from the regions of Denmark to GM. Symbolized with red square is the percentage of test requisitions from each region out of the total number of requisitions from Denmark (n = 515). Symbolized with yellow square is the number of test requisitions pr. 100.000 citizens in each region.

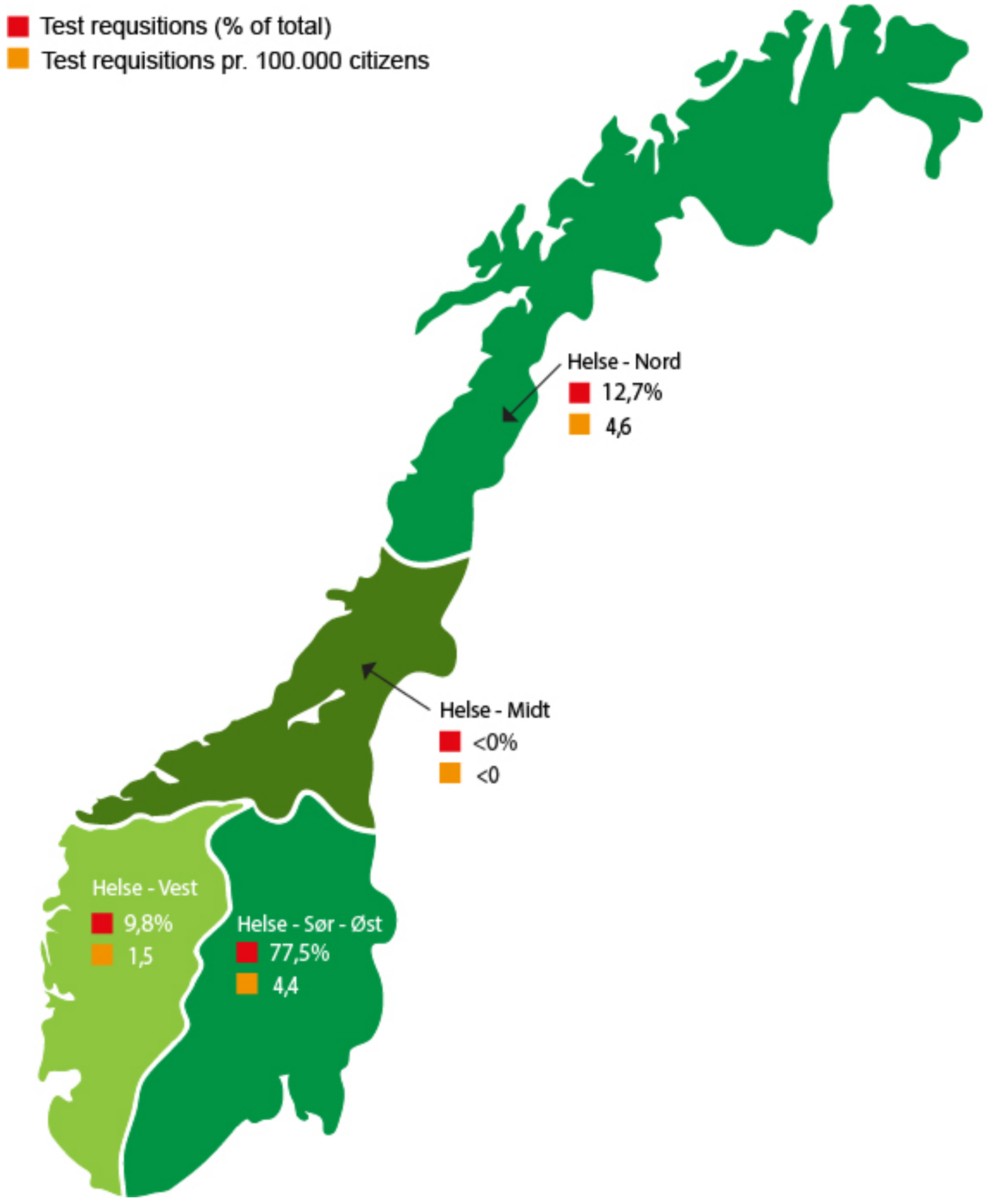

Supplementary figure 3B Schematic overview of the myChoice® CDx requisitions send from the regions of Norway to GM. Symbolized with red square is the percentage of test requisitions from each region out of the total number of requisitions from Norway (n = 173). Symbolized with yellow square is the number of test requisitions pr. 100.00 citizens in each region.

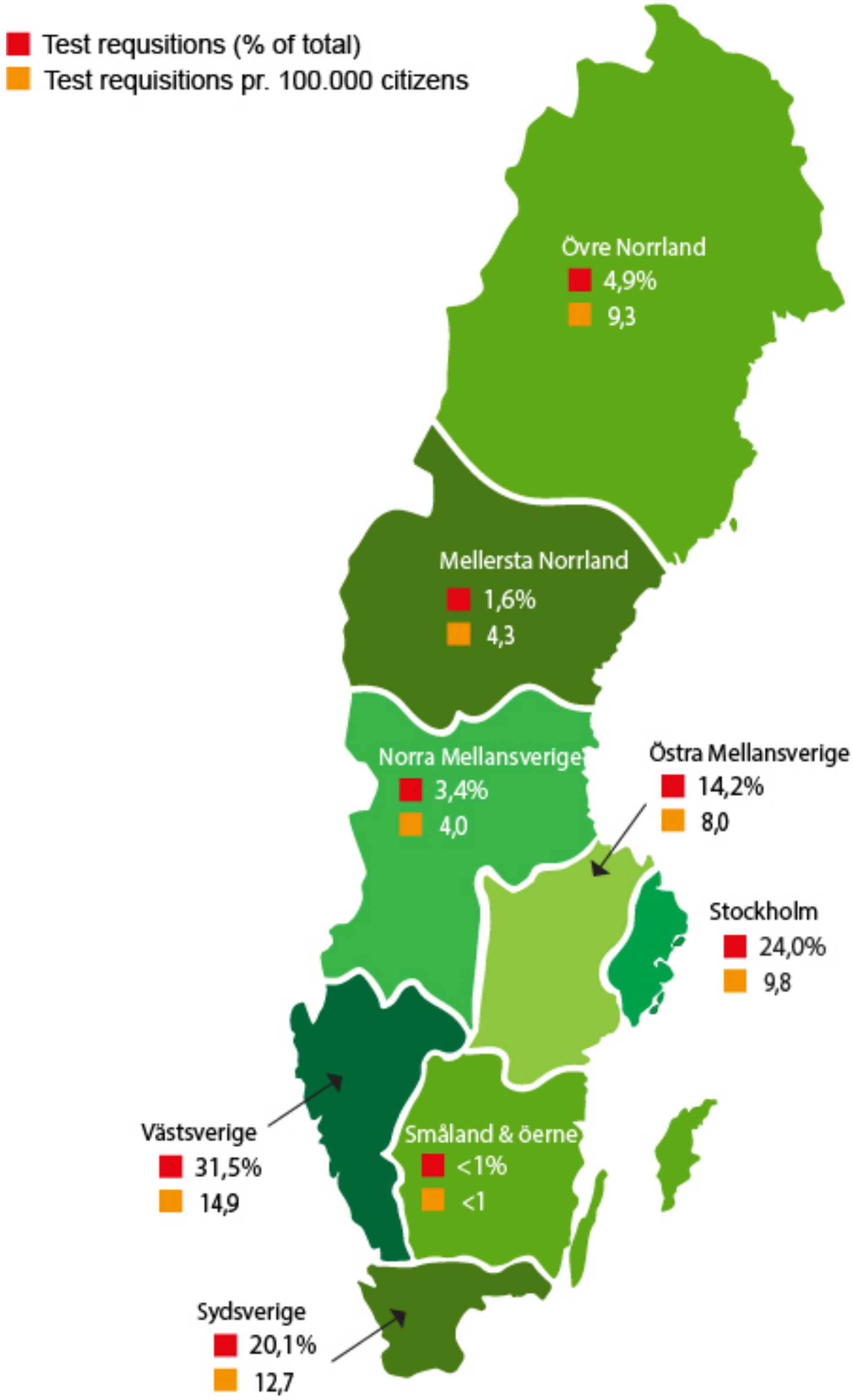

Supplementary figure 3C Schematic overview of the myChoice® CDx requisitions send from the regions of Sweden to GM. Symbolized with red square is the percentage of test requisitions from each region out of the total number of requisitions from Sweden (n = 990). Symbolized with yellow square is the number of test requisitions pr. 100.00 citizens in each region.

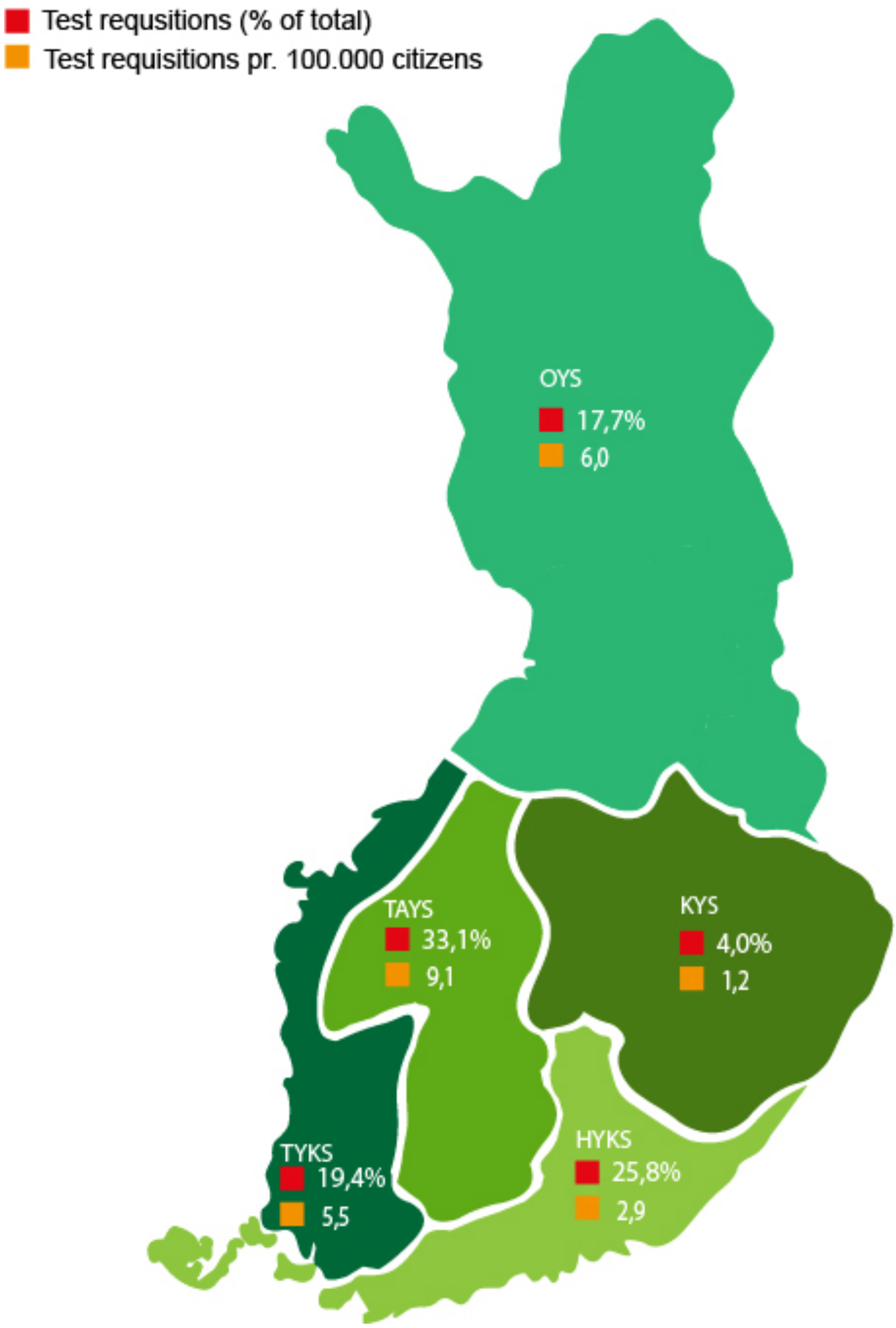

Supplementary figure 3D Schematic overview of the myChoice® CDx requisitions send from the regions of Finland to GM. Symbolized with red square is the percentage of test requisitions from each region out of the total number of requisitions from Finland (n = 248). Symbolized with yellow square is the number of test requisitions pr. 100.00 citizens in each region.
